# Translation of bioethics across cultural borders: exploring the adoption of the four-principles approach in palliative care provision on the Chinese mainland

**DOI:** 10.1186/s12904-025-01733-2

**Published:** 2025-04-10

**Authors:** Shengyu Zhao, Giles Birchley, Richard Huxtable

**Affiliations:** Centre for Ethics in Medicine, Population Health Sciences, Bristol Medical School, University of Bristol, Canynge Hall, 39 Whatley Road, Bristol, BS8 2PS UK

**Keywords:** Chinese bioethics, Palliative care ethics, Translational ethics, Cross-cultural bioethics, Four-principles approach

## Abstract

**Background:**

The four-principles approach is widely incorporated into Chinese curricula and training programs in medicine. Notably, in the training of palliative care practitioners, the literature and the empirical evidence show that the principlist framework appears to be the sole ethical framework taught. However, this framework does not align well with the prevailing cultural practice in China - the family-led decision-making model.

**Methods:**

To better capture the moral and cultural nuances in palliative care provision, 35 practitioners were recruited via purposive and snowball sampling from nine sites in Eastern China for one-on-one semi-structured interviews. All interviews were conducted in Mandarin, the participants’ native language, to accurately reflect the moral claims underlying their clinical practices.

**Results:**

Empirical evidence reveals three key insights. Firstly, families on the Chinese mainland assume a dominant role in medical decision-making, with the power to make decisions regarding care planning and treatment provision on behalf of the patient. This family-led feature is depicted as normative by Chinese HCPs. Secondly, the four-principles approach is the predominant ethical framework recognised by participants. Nevertheless, while the four-principles approach is extensively taught through university courses and occupational training, the family-led decision-making model remains intact in practice and justified by legislation. Finally, a practical solution of a family-first coping mechanism was proposed by the participants, in accordance with the Familistic feature. In this mechanism, the patient is able to make autonomous choices, albeit on the (implicit) precondition of family approval.

**Conclusions:**

Empirical data indicates that the translation of the four-principles approach remains incomplete in Chinese contexts due to its failure to consider the local socio-cultural landscape. The principlist framework overlooks the distinctive conceptualisation of the decision-making unit as a holistic family entity in China and disregards the legal and perceived moral necessity of familial participation in medical decision-making. Consequently, the application of Western bioethics in this context falls short of transcending cultural boundaries, raising critical questions about the validity of conclusions drawn from this theoretical framework.

**Supplementary Information:**

The online version contains supplementary material available at 10.1186/s12904-025-01733-2.

## Introduction

As a well-established ethical framework, the four-principles approach is one of the most widely used frameworks in the field of bioethics [1]. As its name suggests, the four-principles approach encompasses four fundamental ethical principles that healthcare practitioners are encouraged to balance in decision-making: respect for autonomy, nonmaleficence, beneficence, and justice [2].

This Western-originated framework has been extensively adopted in Chinese medical education, including higher education curricula, training programs for registered professionals, and evaluative criteria for both clinical practice and research [3, 4]. In palliative care, in particular, the prominence of the four-principles approach is especially noteworthy. Within existing training schemes, this principlist framework is the sole ethical framework introduced in detail to practitioners [3, 5]. However, the key cultural feature of Chinese society - the family-centred decision-making model - appears to be at odds with this approach. In palliative care settings on the Chinese mainland, decision-making is often undertaken by the family on behalf of the patient, which is perceived as a breach of the individual’s right to autonomy under the principlist framework [6]. Consequently, the practice of Chinese healthcare professionals (HCPs) in permitting family-led decision-making appears ethically problematic when assessed through a principlist lens.

This paper seeks to explore the interaction between the four-principles approach and Chinese cultural norms through the theory of translational ethics. Translational ethics refers to the strategies, plans, and practices involved in applying bioethical theories to clinical practice and vice versa. Its overarching goal is to bridge the gap between theory and practice, facilitating the development of theoretically grounded and pragmatic solutions to real-world ethical challenges [7]. A critical component of translational ethics is the contextual understanding of ethical issues, which includes examining their socio-cultural, economic, and legal dimensions [8]. This paper investigates how the Western-born four-principles approach transcends cultural boundaries and engages with local contexts in China. In-depth interviews with Chinese palliative care practitioners were conducted to examine the practical implications of the four-principles approach at the bedside.

It needs to be highlighted at the beginning that this paper does not primarily focus on solving the ethical question of whether families should formally be stakeholders in medical decision-making. Rather, it aims to investigate the factors that conflict with the full implementation of the principlist framework in the socio-cultural context of the Chinese mainland and the indigenous moral justifications that underpin these conflicting factors.

## Methodology

This paper is based on an empirical bioethics project that aims to map the current landscape of palliative care ethics in Mainland China. The project adopts the three-phase Bristol Framework [9]: *mapping*, *framing*, and *shaping*. The *mapping* phase involves surveying the landscape of the topic using literature reviews, *framing* involves exploring understandings within practice using social science research methods, and the final *shaping* phase constructs recommendations based on a process of reconciling the previous two stages using an empirical bioethics method of reflexive balancing [9].

The empirical evidence presented in this paper is derived from the second phase - *framing* - which focuses on investigating clinical practice through qualitative research methods. This paper identifies gaps between the realities of clinical practice and the values applied to it, highlighting discrepancies that will need to be reconciled in a subsequent phase of research.

This paper is positioned at a transitional stage between the *framing* and *shaping* phases, where the research begins to move from the empirical findings to ethical recommendations. The primary goal at this stage is to uncover the underlying factors that influence the translation of the four-principles approach in Chinese contexts. The findings at this stage are fundamentally derived from qualitative research methods, as introduced in Method section, and will serve as the foundation for the forthcoming *shaping* phase. It is important to emphasise that normative conclusions have not yet been reached. The claims and observations emerging from the *framing* phase may carry normative implications. Nevertheless, as ongoing developments, these claims and observations should not be regarded as fully formed normative recommendations.

## Method

To investigate the practical implications of the four-principles approach in Chinese contexts, empirical data was collected from frontline Chinese HCPs. For the purpose of this research, HCPs are defined as follows:

- **Clinical professionals**: This category includes clinicians, specialists, therapists, nurses, and others working in hospital settings and provide direct patient care. Both publicly and privately funded hospitals were included in the study. To account for the range of medical professionals involved in palliative care, participants may include specialists from geriatrics, oncology, and other related departments.
- **Public health practitioners**: This group consists of general physicians and others not based in hospitals but working in community clinics, hospices, or care homes.
- **Other supportive roles in palliative care teams**: This includes, but is not limited to, (medical) social workers, volunteers, psychologists, and other supportive personnel.

As an empirical bioethics study, this project aims to uncover the ethical nuances embedded in the practice of palliative care on the Chinese mainland using qualitative methods. Given the profound influence of culture on local morality, semi-structured interviews are deemed an appropriate approach, as they are context sensitive [10]. The use of a flexible, revisable question list allows for the emergence of unanticipated ethical challenges, discussions, and reflections from participants. This adaptability helps minimise potential Western-centric biases that may arise from the persistent reliance on Western ethical frameworks.

Given the limited number of palliative care practitioners on the Chinese mainland, participants were recruited using purposive and snowball sampling. As palliative care remains an emerging specialty in the region, the pool of eligible practitioners is relatively small. To facilitate effective recruitment, invitations were sent exclusively to established palliative care teams. Additionally, members of these teams were encouraged to share the recruitment information with colleagues or other professionals they deemed qualified to participate.

Each selected participant received a participant information sheet (Supplementary Material 1), which provided details about the study and interview process. After obtaining informed consent, participants took part in one-on-one semi-structured interviews, following the interview topic guide provided in Supplementary Material 2.

According to Malterud et al.’s [11] information power model, an exploratory research project on a specific topic does not require an extensive sample size. Based on insights from studies on previous empirical bioethics studies [9, 12], a minimum target of 30 participants was initially set, with the possibility of including additional participants if feasible. Ultimately, 35 participants were recruited from nine sites in Eastern China.

To better capture cultural nuances, all interviews were conducted in Mandarin, the native language of the participants and the lead researcher. While several dialects coexist alongside Mandarin in China, particularly in the four Southeastern sites, the use of dialects was not considered for three reasons. First, the lead researcher lacked proficiency in these dialects. Second, participants did not exhibit any difficulties in communicating using Mandarin. Finally, and most importantly, the participants’ previous ethics training had been conducted in Mandarin, making them more accustomed to discussing ethical issues in this language. Therefore, Mandarin was considered the most efficient medium for capturing and documenting the cultural and moral nuances relevant to this study within the context of the Chinese mainland.

### Analysis

After empirical data collection, the interview transcripts were subjected to thematic analysis to identify recurring ethical challenges encountered by participants. To ensure methodological rigor and analytical comprehensiveness, Braun and Clarke’s six-phase framework [10] was employed, comprising: (1) familiarisation with the data, (2) generating initial codes, (3) constructing themes, (4) reviewing themes, (5) defining and naming themes, and (6) producing the final analysis report [10].

The initial coding and theme generation were conducted by the lead author, a native Mandarin speaker, who was able to directly interpret participants’ moral claims within their original linguistic and cultural context. This linguistic proficiency was particularly advantageous in capturing subtle moral nuances and culturally embedded ethical concepts that may not be easily translatable into English.

For this study, three key themes emerged regarding familial participation in decision-making within palliative care: (1) the decisive role of the family, (2) the epistemic recognition of the four-principles approach, and (3) the family-first coping mechanism. As illustrated in Results section, these themes collectively underscore the persistence of family dominance in palliative care decision-making on the Chinese mainland. While awareness of the four-principles approach was reported among participants, its translation into practice diverged significantly from theoretical expectations.

A well-documented critique of thematic analysis is its susceptibility to researcher subjectivity, which may introduce interpretative bias into the analytical process. Given the lead author’s positionality as an ‘insider’ within the Chinese ethical environment, their contextual familiarity facilitated a deeper interpretive understanding of participants’ ethical reasoning. However, to mitigate potential bias - whether Western-centric or Chinese-centric - the coding framework and thematic analysis underwent independent review and cross-validation by the second and third authors, neither of whom have direct cultural ties to China. This external validation process enhanced analytical neutrality and strengthened the credibility and reliability of the findings.

### Reflexivity

As a Chinese PhD student specializing in bioethics in the UK, the lead researcher’s background extensively overlaps with the focus of this study. Consequently, concerns regarding subjectivity bias are understandable and expected. A common response to such concerns is that individual subjectivity should be neutralised during the analysis and reflection process, ensuring that the researcher minimises their influence to maintain academic rigour [13]. However, it is arguably not feasible to completely eliminate the impact of subjectivity from studies involving interpersonal interactions [14, 15]. Instead, the lead researcher’s engagement with the data and analysis actively shapes the production of research outcomes [16–18]. Therefore, in this study and paper, it is undeniable that the lead authors’ personal interests and experiences play a role in motivating and directing the process of data collection, analysis, and reflection.

In this project, the integration of the lead researcher’s subjectivity should be regarded as an advantage, as it enhances the contextual reflexivity of the study [19]. One of the primary reasons for the limited presence of English-language literature on bioethical studies on the Chinese mainland is the lack of familiarity with local languages and cultures. Being an outsider can inevitably hinder a researcher’s ability to comprehend and interpret the implications of indigenous moral codes. In contrast, the lead author possesses the linguistic and cultural knowledge necessary to decode ethical frameworks on the Chinese mainland through their personal experiences. In this sense, subjectivity should not be seen as a limitation, but rather as an enhancement of the analytical process.

Nonetheless, while subjectivity can positively contribute to the research, inappropriate bias must not be tolerated. Therefore, as outlined in Analysis section, double coding and external review were implemented to ensure that any subjectivity is monitored and constrained.

### Ethics approval

Ethical approval was primarily granted by the host institution of this research, the Faculty of Health Sciences Research Ethics Committee, University of Bristol (reference: 12319).

As advised by the Faculty Ethics Committee, local ethics committees on the Chinese mainland were also consulted regarding their requirements for ethics approval. Since this project does not involve the use of human tissues or clinical trials, the respective local sites reported that no additional ethics approvals were required.

## Results

As introduced in Method section, a total of 35 participants were recruited. In general, female participants constituted the vast majority, with only five male participants included in the study.

Regarding the participants’ roles, the distribution across the four groups - physicians, nurses, social workers, and volunteers - was relatively balanced. Among the clinical professionals, there were 13 physicians and 8 nurses. Additionally, the number of volunteers (seven) and social workers (seven) were equal.

The participants were predominantly middle-aged (above 30 years old) and comparatively well-educated, holding at least a bachelor’s degree or higher. Moreover, two-thirds of the participants had less than five years of experience in palliative care, which aligns with the recent development of this practice on the Chinese mainland.

Further details are provided in the Table 1.

**Table 1:** Participants’ Information

		**Total**
**Gender**	Male	5
	Female	30
**Age**	< 30	4
	30–40	14
	40–50	11
	> 50	6
**Highest education level**	Unknown	3
	BSc/Bmed	14
	MSc/Mmed/PhD	18
**Role**	Physician	13
	Nurse	8
	Social worker	7
	Volunteer	7
**Year of experience**	< 1 year	3
	1–3 years	9
	3–5 years	12
	> 5 years	11

Drawing on empirical evidence, direct care on the Chinese mainland continues to be predominantly family-led. Based on the interviews conducted, while there is general awareness of the four-principles approach and the respect for patient’s autonomy in particular, the interaction between HCPs, the family, and the patient still appears to be at odds with this framework. To address this epistemic contradiction between the emphasis on the individual patient and family autonomy, family meetings are employed as a mediating mechanism. However, within this structure, the central role of the family remains largely intact, even as efforts are made to uphold the patient’s autonomy.

### Theme 1: The decisive role of the family in medical decision-making

From a sociocultural perspective, the interaction between the patient and their family in palliative care reflects a strong Confucian Familist orientation. In decision-making, the family often assumes a decisive role throughout the entire care provision process [20–24]. Particularly, in cases of disagreement between the family and the patient, the family appears to hold greater authority, granting them the power to override the patient’s wishes.

‘*This grandpa always wanted to go home [and pass away at home], yes, he always wished to go home. However, in the end, it didn’t happen. He wasn’t able to go back because his family firmly refused to give permission.*’ [N1, nurse]

‘*After recovering from an acute lung infection that led to respiratory failure, the patient told me that if he experienced another severe respiratory failure and the doctors judged that it would be hard for him to recover, he did not want to go to the ICU or be intubated. He had expressed this to me. About a month later, when his condition worsened, his daughter was extremely distraught, and his son insisted on doing everything possible to save him, meaning they wanted intubation. In the end, the patient was intubated in the ICU, extubated, then re-intubated, and later underwent a tracheotomy. He passed away shortly after, likely enduring a very painful end. He was already 97 years old.*’ [P1, geriatrician]

As illustrated by the quotations, throughout the care process, the patient appears to occupy a relatively weak position, while the family holds ultimate authority in decision-making. When reflecting on these experiences, participants described familial involvement as normative - it is perceived as both natural and necessary for HCPs in China to include the family in caregiving. Even when recognising these situations as ethically complex due to the underrepresentation of the patient, participants did not perceive family-led decision-making as morally inappropriate.

‘*It’s just that, if the family can’t agree, we can’t really force them to comply. We just keep talking, trying to communicate over and over. But if we [the family, the patient and the HCPs] still can’t reach an agreement, then there’s nothing else we can do. It just feels like we’re kind of powerless in that situation.*’ [N1, nurse]

‘*If the patient is conscious, of course the decision should be made by the patient themselves. But what if the family strongly opposes that decision? That’s one of the major dilemmas we face in clinical practice.... If it’s truly impossible to agree, then we might end up compromising. I understand that someone will have to give way, and that often means listening to the family’s wishes.*’ [P1, geriatrician]

In fact, all interviewed HCPs demonstrated, to varying degrees, a tendency to prioritise the family’s decisions, even in cases of disagreement with the patient. Participants justified this practice by framing it as the most appropriate solution within the sociocultural context.

### Theme 2: The epistemic recognition of the four-principles approach

Despite the strong familist orientation on the Chinese mainland, the four-principles approach is explicitly recognised here as an ethical framework. It was the only framework directly mentioned by participants when asked to describe their understanding of bioethics.

‘*What I’ve been trained in ethics is just the four-principles approach. There are four ethical principles: autonomy, nonmaleficence, beneficence, and, um, justice.*’ [N2, nurse]^1^

Although some participants did not demonstrate a comprehensive understanding of the four-principles approach, the principles of nonmaleficence, beneficence, and respect for the patient’s autonomy were individually highlighted during discussions. Among these, respect for autonomy was most explicitly identified as one of the ‘golden rules’ of palliative care.

‘*For palliative care, it emphasises that “It’s my life, it’s my decisions.”*’ [P2, geriatrician]

‘*Life itself should be governed by one’s own autonomy, in my view. Palliative care highly values and respects each individual’s right to make their own choices. This is why we aim to return the decision-making power over one’s life to the individual, rather than to doctors or family members.*’ [S1, social worker]

Notably, among the four principles, adherence to individual patient autonomy appears to be afforded ethical privilege in the context of palliative care on the Chinese mainland. As indicated in the quotations above and the case discussions in the interviews, Chinese HCPs often interpret the principle of respect for patient autonomy as the prioritisation of the patient’s wishes and opinions over those of the family in caregiving. One possible reason why participants emphasise this individualistic concept is that it remains relatively novel to them. Highlighting its significance may help reinforce awareness and promote its practice.

The acceptance of, and adherence to the four principles, particularly the principle of respect for autonomy, at the cognitive level, indicate a partial accomplishment in the translation of the four-principles approach within Chinese contexts. However, there is a risk of over-emphasising the primacy of autonomy. Some defenders of this position, such as Gillon [25], argue for its pre-eminence, whereas Beauchamp and Childress [2] adopt a more nuanced perspective. This misinterpretation suggests that Chinese HCPs may have a simplified or incomplete understanding of the four-principles approach.

When asked about the source of their ethical knowledge, participants indicated that their understanding of the four-principles approach was primarily acquired through higher education and occupational training programs.

‘*In the degree program curriculum, there’s certainly medical ethics included. Right now, I’m also involved in teaching what we refer to as “professional competencies” [course], which incorporates modules on medical ethics. Then there’s what we call “ideological and political education” unit [in higher education curriculum and training], or simply “thought and politics”, and that also contains some ethical elements.*’ [P3, oncologist]

These responses demonstrate that awareness of the four-principles approach is widespread among Chinese HCPs. This finding aligns with the recently published *Bluebook of Palliative Care Development in China (2019 - 2020)*, which highlights the ongoing development of palliative care on Chinese mainland [4]. Fundamentally, ethics training schemes on the Chinese mainland - a pivotal component in the translation of bioethics - are structured in alignment with principlism.

Compared to the family-led decision-making model discussed in the previous section (Theme 1 section), the recognition of the four-principles approach does not appear to be effectively translated into practice, where the family remains the key stakeholder. While participants consistently acknowledged their knowledge of bioethics and expressed consensus on the value of patient autonomy, the patient often remains ‘invisible’ in actual clinical settings.

Rather than respecting the decision of the individual patient that respect for autonomy implies, the decisive role of the family was explicitly affirmed by participants during interviews. When conflicts arose between the family’s preferences and the patient’s wishes, participants frequently reported siding with the family. One justification for this practice was the perception that the patient, due to their vulnerability, was deemed unable to make autonomous decisions.

‘*Here, patients are generally in a vulnerable position. Most of the time, HCPs tend to comply with the family’s wishes even the patient resists, as the patient is often too vulnerable because of the illness or age. As a result, it is usually the family members who make the key decisions.*’ [P1, geriatrician]

This statement might partially justify the power of the family when the patient is incapable of making decisions for themselves. For other scenarios, the familial decision-making seems to be validated through legislation.

‘*It is a legal document, so the surrogate has the right to decide. Therefore, I must ask for his or her opinion. Even when the patient is conscious, or in other words, mentally capable, we still need the surrogate’s permission to provide any invasive treatments. This is because firstly required by law, and secondly the family also has the right to know.*’ [N2, nurse]

This legal document is verified in the *Civil Code of the People’s Republic of China* (the *Civil Code*), in which Article 1219 clearly states that the family can provide informed consent for the patient if the HCPs deem appropriate. In this sense, the family is granted with legal power of substitute decision-maker, even when the patient is competent to make the decision.

Whether based on legal authority or perceived patient incompetence, the family in Chinese contexts appears to assume a justified surrogate role in medical decision-making. This dynamic closely aligns with Confucian Familism, which grants the family precedence over the individual. Consequently, in palliative care practice, a family-led model remains morally acceptable within Chinese cultural and ethical frameworks.

However, during interviews, HCPs consistently reported experiencing confusion, struggle, and pressure when navigating conflicts and disagreements between the patient and their family. In particular, when patients were subjected to avoidable suffering and pain due to familial decisions, HCPs expressed a profound sense of moral unease.

In the aforementioned scenarios, it seems that local Chinese moral norms of Familism and Principlism are not well integrated into the practice. This conflict underscores the limitations of the four-principles approach in effectively addressing the role of the family in Chinese palliative care practices. It highlights that the justification and action guide generated by this framework is rendered ineffective in these contexts, as the role of the family is not adequately addressed within the principlist framework - at least as understood by these participants.

### Theme 3: A family-first coping mechanism

Despite the ethical tensions between the family and the patient, a coping mechanism appears to have emerged within the family-oriented model. Patient autonomy seems to be implicitly granted through familial approval, allowing a patient-driven decision-making process to align with the principlist framework.

‘*Yes, because we have a very strict “gatekeeping rule” for admitting patients to our ward. During the initial consultation, I clearly explain to the family members the logic behind palliative care. The fundamental principle of my work is to provide care in the way the patient desires. So, if the family wishes to hide the truth from the patient until their last moments, we cannot accept such patients*’ [P4, oncologist]

‘*We have family meetings.... In situations where the patient is firmly opposed to something but the family is equally adamant about going forward, we sit down to discuss it together. I’ll bring up the patient’s viewpoint, and ideally, the patient will join the family meeting so everyone can talk it through. We ask whether the family is willing to honour the patient’s wishes or if they think their own concerns should take precedence. Our aim is to guide them to see the patient’s needs. If, in the end, the family still won’t respect the patient’s preferences, we go back to the patient and ask if they want to change the authorisation. Since the authorisation letter can be modified as long as the patient is still competent, they can choose to appoint anyone - usually a close family member.*’ [N3, nurse]

Patients in these cases were able to make decisions according to their wishes. However, the family is not entirely excluded from the decision-making process. Instead, familial consent appears to function as a prerequisite for the patient’s exercise of autonomy in palliative care. The patient’s wishes are respected only with the family’s acquiescence.

As a result, the principle of respect for patient autonomy in Chinese palliative care settings is practised conditionally - the patient receives a form of quasi-respect that remains contingent upon family cooperation. While the ultimate act of respecting patient autonomy in clinical settings may resemble Western practices, the underlying conditions differ significantly.

## Discussion

The misalignment between training in the four-principles approach and clinical practice in palliative care suggests that the translation of the principlist framework is still far from guaranteed. However, this should be viewed as a failure of completion, rather than as an outright failure of translation. Labelling the process as incomplete aligns with a fundamental characteristic of bioethical reasoning - its iterative nature. As illustrated in many bioethical methodologies, such as reflective equilibrium and reflexive balancing, the justification of an argument involves a dynamic, iterative process [26]. In sound reasoning, multiple iterations between empirical and/or theoretical evidence often occur before a final conclusion is reached. Therefore, rather than declaring the failure of the four-principles approach, the current situation on the Chinese mainland appears to reflect an evolving process in which practical realities bring previously underacknowledged elements into consideration.

As evidenced in Theme 1 and Theme 3 sections, while the HCPs demonstrate a degree of familiarity with the four-principles approach, clinical practice remains predominantly family-led. Theme 3 section, in particular, illustrates how Chinese HCPs attempt to reconcile principlism with deeply embedded local moral norms. Despite the theoretical awareness of principlist bioethics, the familist core of Confucian ethics continues to dictate clinical decision-making. In practice, the family retains a dominant decision-making role, often operating discreetly ‘behind the veil’ of formal patient-provider interactions. Family authorisation is frequently conducted under the table and outside official discussions with the patient. Only after securing familial approval can the principlist informed consent procedure be formally initiated with the patient. This approach reflects a hybridised ethical practice, in which principlist autonomy is performed conditionally, contingent upon prior family endorsement.

This section elaborates on three primary reasons for the participation of the family in caregiving and the unique coping mechanism to the four-principles approach in Chinese contexts: (1) categorisation of the decision-making unit, (2) perceived moral necessity, and (3) the legal necessity of familial involvement. Given these three reasons, it becomes evident that the dominance of Familism in China accounts for the apparent failure to effectively translate Principlism into this context.

### Categorisation of the decision-making unit

A primary reason for the incomplete translation of the four-principles approach is the inadequate acknowledgment of the distinctive categorisation of the care recipient in Chinese contexts.

On the Chinese mainland, as in many other Asian societies, the individual occupies a less central role in the social system. Instead, the family is considered the fundamental unit of society [27]. As Fan ([28], p.74) points out, under the influence of Confucian Familism, *‘it is the family, rather than separate individuals, that constitutes the ultimately autonomous unit of decision-making from the rest of society’*.

In Chinese contexts, the family often refers to a three-generation household comprising grandparents, adult parents, and children cohabiting under the same roof [29]. These three generations are united as a singular, collective unit, reflecting the essence of Chinese Familism, which is deeply rooted in Confucian philosophy.

The indivisibility of the family is intrinsic to Confucian Familism, which upholds that the moral goal of the family is collective and cannot be reduced to the well-being of individual members. As Fan ([29], p.3) states:

‘*the good of the family ought to be pursued by every family member. It [Confucian familism] takes the good of the family to be irreducible to the good of each individual family member.*’ ([29], p.3).

As a mechanism to safeguard family integrity and promote the family’s best interests, each individual family member is morally obliged to assume the role of caregiver whenever another member is in need. This duty contributes to the collective good of the family through the enhancement of individual well-being.

While one might argue against the inseparability of family good, it is important to clarify that family interests cannot be equated to the simple aggregation of individual interests [29]. However, the best interests of each family member inevitably influence the achievement of the broader family good. In this sense, individual interests and family interests in Confucian ethics are positively correlated. Thus, the improvement of individual well-being is essential to the promotion of family good.

This holistic conceptualisation of the family profoundly influences the notion of privacy in Chinese culture. In contrast to Western perspectives, where individual privacy is considered a fundamental human right, in Chinese contexts, privacy is defined within the domain of family matters [24]. Within this framework, issues and information shared within the family are regarded as private, whereas interactions between the family and ‘outsiders’, such as HCPs, are perceived as non-private, social relations. Individual privacy is thus subsumed under familial privacy, with no distinct consideration for the individual apart from the family unit. Accordingly, familial privacy, shaped by Confucian Familism, reinforces the collective identity of the family, whereby individual members are not isolated as autonomous subjects [24, 29].

In addition, the family not only functions as a unit of privacy but also establishes the ethical boundaries of professional conduct. In the context of hospital visits, interactions between HCPs and the family are classified as non-private relationships, in which HCPs engage with the family as an indivisible whole. Conversely, intra-familial interactions, including those involving the patient, are considered private matters, discouraging HCPs from intervening in what is perceived as ‘family business’. In this sense, the familial unit delineates the ethical limits of HCPs’ professional intervention.

While the four-principles approach acknowledges the role of the family, its moral status within this framework appears to be significantly different. In the principlist framework, the autonomous decision-making unit is the individual. Beauchamp and Childress ([2], p.99) have repeatedly stated that the four-principles approach is *‘not excessively individualistic to the neglect of the social nature of individuals’*. Nevertheless, it is undeniable that this framework remains fundamentally individual-centred. The four-principles approach designates the individual as the primary locus of care. As Beauchamp and Childress assert, the individual patient is granted *‘the fundamental ethical and legal right to know and decide’* ([2], p.119). Consequently, decision-making in caregiving is driven by the interests of individuals [2].

The emphasis on individuality extends further to the interpretation of the four principles. Although Beauchamp and Childress [2] clarify that the principle of respect for autonomy does not hold moral priority over the other principles, Gillon ([25], p.310) designates it *‘primus inter pares’* (‘first among equals’), as it establishes the individual-centric tone of the framework. In the seminal work *Principles of Biomedical Ethics*, the principle of respect for autonomy is explicitly applied to the *‘individual’* ([2], p.99), *‘person’* ([2], p.100), or *‘actor’* ([2], p.102), each described in the singular form, possessing the capacity for *‘self-governance’* ([2], p.100). Furthermore, the principles of beneficence and nonmaleficence are primarily framed around the individual patient [25], with no formal recognition given to the family as a stakeholder in ethical decision-making.

Within the principlist framework, while the family may assume an authoritative role in caregiving, this role is conditional. Specifically, the family can act as a surrogate decision-maker with presumptive authority when *‘patients are not autonomous or are doubtfully autonomous’* ([2], p.139), for two reasons. First, the family is expected to have *‘a sufficiently deep familiarity with the patient that the particular judgment made reflects the patient’s views and values’* ([2], p.140). Secondly, the family is considered the primary decision-maker because *‘they usually have the deepest interest in protecting their incompetent members’* ([2], p.193). However, this entitlement is conditional, and any breach of these conditions may lead to the restriction or termination of the family’s authority. As such, according to the principle of respect for autonomy, the family’s decisions must align with the patient’s precedent autonomy. The principles of nonmaleficence and beneficence further require the family to avoid potential harm and act in the patient’s best interests when serving as a surrogate decision-maker. Meanwhile, HCPs bear the responsibility of monitoring the family’s conduct and disqualifying the family as a decision-maker if this is judged necessary to *‘shield incompetent individuals from family members who care little or are caught in conflicts of interest’* ([2], p.193).

In short, within the four-principles approach, the family is framed in two distinct roles: either as a secondary backup when the patient lacks the capacity for autonomous decision-making or a potential threat to the patient’s autonomous decision-making. In both cases, the family is relegated to a secondary and often supplementary role in care decisions.

In this vein, the distinctive moral status of the family appears to differ in Confucianism and principlism. In Confucian ethics, the family holds a foundationalist moral status, serving as the foundation upon which Confucian moral norms are constructed from a family-centred perspective. In contrast, the four-principles approach positions the family as peripheral, with its role justified only in relation to the interests of the patient.

When these two conceptualisations of the same role collide, the moral status of the family becomes unsettled due to contradictory moral expectations. This asymmetry in moral weighting between the family and the individual in Confucian and principlist frameworks creates a significant epistemic mismatch, which obstructs the application of the four-principles approach within the Chinese cultural context. In clinical practice, the Confucian perception of the family as a unit of privacy presents a significant challenge for HCPs seeking to uphold biomedical ethical standards. While the four-principles approach advocates a patient-centred model, which enables professionals to intervene based on respect for autonomy, the Confucian emphasis on familial privacy restricts their ability to act without familial consent. Consequently, HCPs often find themselves constrained by cultural expectations that define ethical boundaries not only in terms of patient rights but also familial authority and discretion over medical decisions.

Since, in the two ethical frameworks, the outstanding distinction concerns who or what it is that counts as an autonomous unit [2, 28], a possible solution, which would enable the four-principles approach to be accommodated in Chinese contexts, might involve replacing the individual with the family as the central unit of concern in principlism. Yet, this straightforward substitution cannot adequately capture the collectivist and relational dimensions intrinsic to Confucian Familism when applying the four-principles approach. Fundamentally, the principlist framework maintains the individual as its central focus. As a result, the pursuit of family integrity is absent from its moral ideal.

This fundamental conflict over the conception of the ethically salient decision-making unit between Confucian Familism and the individual-centred four-principles approach presents a major challenge to the translation of the four-principles approach into Chinese contexts. The divergence in conceptualising the care recipient - whether as the individual patient or as the family as a whole - resonates with the epistemic inconsistency rooted in the two frameworks as identified in the empirical evidence. As Beauchamp and Childress [2] do not primarily emphasise the moral status of the collectivist family, the incompatibility between these two philosophies undermines the efficacy of the four-principles approach in this cultural environment.

### The fulfilment of moral duty by the family

A second major obstacle to the full integration of the four-principles approach in Chinese medical ethics is its insufficient recognition of the moral primacy of familial obligations in Confucian ethics. In Confucianism, the pursuit of collective familial well-being serves as a moral ideal, structuring ethical responsibilities around role-based duties [20, 29]. Each family member is assigned specific obligations that prioritise interdependence over autonomy, reinforcing a framework in which moral agency is distributed across the family unit rather than located in the individual alone [24]. This collectivist orientation stands in contrast to the principlist assumption that ethical obligations should be individually determined, creating an inherent epistemic conflict when attempting to apply the four-principles approach in Chinese clinical settings.

In Chinese palliative care, caregiving responsibilities are deeply intertwined with Confucian filial piety, particularly within the grandparent-parent dyad, where adult children bear both moral and social responsibility for the care of their ageing parents. Unlike in Western contexts, where caregiving is often voluntary and framed as a personal choice [30], Confucian ethics conceptualises it as a hierarchically enforced, non-negotiable moral duty. Filial piety extends beyond practical caregiving, which includes financial and emotional support [22, 31], to encompass an ethical commitment to ‘*deference (placing one’s parents’ interests before one’s own), obedience, care, and non-abandonment*’ ([21], p.496). In Confucianism, filial piety is not merely a virtue but a foundational ethical principle, institutionalised within Chinese moral philosophy and societal expectations. Confucian classics repeatedly emphasise the primacy of filial piety: Wang ([32], p.250) describes it as *‘the root of humanity and morality’*, while the *Fireside Talk at Night* asserts that *‘of all the virtues, filiality comes first’*. This deep-seated moral imperative not only shapes interpersonal relationships but also permeates medical decision-making, thereby challenging the applicability of the four-principles approach, which assumes autonomous self-determination as the default ethical framework.

While modern medical advancements have shifted hands-on caregiving responsibilities from families to clinical professionals, the ethical obligations of filial piety remain central to medical decision-making in the Chinese contexts. Instead of providing direct physical care, adult children on the Chinese mainland fulfil their moral duty through advocacy and decision-making, ensuring that every possible treatment option is pursued [33]. Within this context, proactive medical decision-making becomes a symbolic expression of filial devotion, reinforcing the adult child’s moral standing within the family hierarchy. Consequently, the adult child assumes the role of a moral agent on behalf of the patient, a conceptual shift that fundamentally contradicts the four-principles approach, which presupposes individual self-governance as the foundation for ethical legitimacy.

Given the fundamental role of filial piety, this principle frequently takes precedence over other ethical obligations. Consequently, in Chinese palliative care, medical ethics - as a form of professional ethics - is often rendered secondary to filial obligations. This moral hierarchy places an expectation on HCPs to respect the family’s fulfilment of filial piety, unless such practices explicitly violate hospital regulations or legal boundaries. Only after accommodating filial obligations can HCPs fully act upon their professional ethical duties. Failure to uphold this cultural norm risks accusations of moral failure, subjecting HCPs to social stigma for undermining filial responsibilities.

In their recent works, Beauchamp and Childress [2, 34] acknowledge the family-centred care model as an enrichment of moral pluralism. The Familism underpinning this model is categorised as a form of *‘particular morality’*. This categorisation is based on Beauchamp and Childress’s advocacy of common morality theory. In short, common morality refers to *‘the set of universal norms shared by all persons committed to morality.... It’s applicable to all persons in all places, and we *[the authors]* appropriately judge all human conduct by its standards’* ([2], p.3). The four principles in the principlist approach belong to common morality and are therefore granted moral authority over all communities. A distinctive feature of common morality is that its moral principles are *‘abstract, universal, and content-thin’* ([2], p.5). In contrast, ‘particular morality’ refers to morality that is not universally shared. While derived from common morality, particular moralities embody *‘concrete, non-universal, and content-rich norms’* within a specific context ([2], p.5). These particular moralities differ in their level of specificity - or, in other words, their scope of application. Nevertheless, all particular moralities must be aligned with common morality, otherwise, their norms are not morally justified if they violate norms in the common morality ([2], p.5) ([35], p.1297).

In this vein, Familism, as a form of particular morality, must be consistent with the four-principles approach to maintain its moral validity. Furthermore, given cultural variations, Familism can modify the concrete practices of the four-principles approach to align with the local Confucian ethos. This process is termed *‘specification’* by Beauchamp and Childress, allowing for *‘adding content’* to common morality ([2], p.17). Through this process, particular moralities can *‘reduce the indeterminacy of abstract norms and generate rules with action-guiding content’* for their adherents by *‘narrowing the scope of the norms’* ([2], p.17). Thus, in the specific context of China, Familistic practices are expected to first adhere to principlist guidelines. Accordingly, Confucian Familism could refine these principles by specifying them to determine *‘who should be the primary moral agents and what their roles should be in elder care within a given society’* ([34], p.172).

It becomes apparent that the duty of filiality does not carry the same moral weight within the principlist framework. Rather, the act of filial piety is regarded as supererogatory - an optional moral practice that *‘exceed[s] what the common morality of obligation demands’* ([2], p.46) from a principlist perspective. This feature appears to be consistent with the moral implication of family caregiving discussed in Categorisation of the decision-making unit section. From a principlist point of view, familial caregiving duties in medical contexts are regarded as voluntary [30] and may even be unwanted if they interfere with an individual’s autonomy or their best interests [2]. In this view, the duty of filial piety is considered subordinate to the four prima facie principles and therefore modifiable, or even removable, due to its optional nature within the principlist framework when practicing in Chinese contexts. This permissible adjustment implies a moral reclassification of the family, shifting its role from a primary subject to a secondary player, while simultaneously isolating the patient from their familial context.

However, classifying Confucian Familism as a form of particular morality and attempting to accommodate it through specification does not sufficiently account for the fundamental differences between Confucian ethics and the four-principles approach. While Beauchamp and Childress describe specification as a process of adding action-guiding content to common morality [2], this assumes that particular moralities derive their legitimacy from common morality. Specification operates as an addition of contextual details without altering the foundational moral structure. Therefore, principlism expects the particular moralities to adhere to a similar, if not identical, epistemic foundation. Nevertheless, Confucian Familism is not merely a culturally specific version of principlism; rather, it presents an alternative ethical paradigm in which moral authority is situated in the family unit rather than the individual. The attempt to ‘specify’ Confucian ethics within principlism preserves the epistemic dominance of the four-principles approach, failing to recognise that the Confucian model is not a subsidiary moral framework but a distinct system in its own right. Consequently, attempts to specify the four-principles approach within this framework are epistemically misaligned rather than merely incomplete.

It could potentially be argued that Familism is not a focus of Beauchamp and Childress’s theory, so the differences between the two do not present a problem for principlism. However, the omission of Familism from principlism suggests that the latter cannot entirely claim to be founded on common morality. Principlism overlooks or diminishes the distinctive Confucian foundation, particularly the centrality of familial obligation and therefore the duty of filial piety. Despite its ethical primacy in Confucian moral philosophy - where filial piety is regarded as the root of all virtues and the moral cornerstone of interpersonal relationships - this duty is relegated to a peripheral role within the principlist framework. This marginalisation reflects a deeper epistemic gap between the two moral paradigms.

Fundamentally, principlism and Confucianism adopt competing conceptions of the duty of filial piety, which respectively emphasise individual-first as opposed to family-first. While Beauchamp and Childress [2] consistently emphasise that the four principles are placed on an equal moral footing, as one of their prominent defenders, Raanan Gillon, conveys, the core ethos of the principlist framework is founded on the tenet of *‘respect for autonomy as first among equals’* ([25], p.307). Individual autonomy and its actualisation are regarded as the ethical premise of moral practice. Adherence to individual autonomy not only clarifies what morality entails but also helps define the content and criteria of beneficence and nonmaleficence in relation to a particular agent [25]. In contrast, Confucian ethics grants filial piety primacy over all other moral duties, which - without exception - should be fulfilled, before any competing ethical obligations are met [32]. Within this framework, the rights or interests of the individual remain secondary to familial ethical obligations.

These contrasts underscore the absence of explicit recognition of the family as a key stakeholder within the principlist framework. This lack of acknowledgment not only restricts the applicability of the four-principles approach in culturally diverse settings but also heightens the challenges associated with its implementation in clinical practice. As evidenced in Results section, despite theoretical awareness of principlism and a strong emphasis on patient autonomy, direct care continues to align with family-led Confucian ethical norms. The dominance of Familism suggests that, in practice, the moral weight of filial piety often supersedes individual autonomy, reinforcing a fundamental epistemic divergence between principlist theory and Confucian clinical ethics.

The dominance of filial piety in medical decision-making raises critical questions about the process of specification and the broader common morality theory upon which the four-principles approach is built. In particular, this phenomenon invites a fundamental inquiry: can regional moral systems retain their own ethically privileged principles within an otherwise principlist framework, especially when their moral frameworks are founded upon inherently different epistemic foundations?

This challenge extends beyond the question of how to specify moral principles in practice - it calls into question the very source of normativity in ethical systems. The four-principles approach, grounded in common morality theory, assumes that universal moral norms serve as the foundation for ethical deliberation, with particular moralities functioning as contextual specifications of these universal principles. However, the persistence of family-led medical decision-making in Confucian contexts suggests a fundamentally different moral landscape, one that resists full integration into this universalist model.

This epistemic tension gives rise to several critical theoretical inquiries:

1. Can local moral systems possess independent ethical authority if they do not share the epistemic foundations of principlism?
2. If local moral systems can hold ethical authority, does categorising them as particular moralities - without further qualification - oversimplify their normative significance?
3. To what extent can principlism genuinely accommodate moral pluralism, or does it inherently prioritise individual autonomy as a universal moral principle?

These unresolved tensions indicate that the translation of principlism into Confucian contexts requires more than mere specification and adaptation. Rather than simply modifying its application, a fundamental reassessment of how the four-principles approach engages with non-individualistic ethical traditions is necessary. If ethical validity is not solely contingent upon adherence to universalist principles, then the cross-cultural applicability of principlism may demand a deeper structural re-evaluation, rather than superficial adjustments within its existing theoretical framework.

If the ethical primacy of filial piety was to be reconciled with the four-principles approach, then replacing individual autonomy with family autonomy might offer a more culturally attuned model, reinforcing the family’s central role in ethical decision-making. However, this proposal directly challenges the foundational structure of principlism by undermining the distinction between common morality and particular moralities. Without rigorous justification, the translation of the four-principles framework in the Chinese context risks becoming a superficial accommodation - one that merely reframes existing cultural practices using Western ethical terminology. Such an approach would not only dilute the theoretical integrity of principlism but also weaken its reflexivity and practical applicability in cross-cultural settings.

Although attempts have been made in Beauchamp and Childress’s work, *Principles of Biomedical Ethics* [2], to address the allocation of the family’s role within the four-principles approach, their responses remain insufficient and leave key concerns unaddressed. Specifically, two case studies on familist practices are included in Beauchamp and Childress’s discussion on autonomy in that book. However, no formal responses have been proposed to explicitly address these non-Western ethical principles.

The first study, examining Korean and Mexican Americans with a stronger preference for withholding medical information from the patient, was conducted within the United States [36]. Blackhall et al. [36] further explore how acculturation - the process by which outsiders adapt to the local culture - influences perceptions of autonomy and truth-telling preferences among immigrants from familist cultures. In this discussion, the four-principles framework remains intact as the ethical foundation and the starting point of justification. However, this case study and its accompanying discussion do not appear to shed light on the questions posed in this paper, as these inquiries arise from a consistent Confucian cultural and moral environment within the Chinese mainland. In this context, there is no necessity for individuals to adapt to or integrate Western ethical norms in their daily lives. Instead, it is the ethical framework itself that is challenged by practice and, consequently, in need of adaptation.

The other example included in Beauchamp and Childress’s work examines Navajo ethics. This case study, conducted within Navajo reservations, bears greater similarities to the empirical data presented here. However, Beauchamp and Childress [2] do not provide explicit guidance on how Navajo HCPs can integrate the four-principles approach into their clinical practices without disrupting local ethical norms. In short, Beauchamp and Childress’s work does not appear to offer a meaningful referential framework for addressing the ethical dilemmas faced by Chinese HCPs.

In short, the four-principles approach appears to be poorly suited to the Confucian principle of filial piety. It is undeniable that maintaining the principlist framework in its original form will lead to unavoidable dilemmas, in which Chinese HCPs face significant challenges in reconciling these two ethical frameworks within their clinical practice. The emergence of this new ethical dynamic thus calls for further negotiation and investigation into the interaction between the four-principles approach and Confucian ethics on the Chinese mainland.

### The legal necessity of familial participation

The final shortcoming of the translation of the four-principles approach is its lack of accommodation for the legally mandated involvement of the family in palliative care. In practice, legislation sets non-negotiable standards for how the four-principles framework can be implemented. In the Chinese contexts, the legal requirement arises from the procedural norms governing informed consent. As noted in a quotation from Theme 2 section, the family must sign a ‘*legal document*’ [N2, Nurse] appointing one of their family members as surrogate empowered to make decisions on the patient’s behalf. Such documentation is mandated by various Chinese medical laws and regulations^2^. For example, the *Civil Code* expressly states in Article 1219 that

‘*The medical staff shall explain the medical conditions and treatment measures to the patient in diagnosis and treatment thereof. Where a surgery, a special examination, or a special treatment is needed, the medical staff shall explain to the patient the medical risks, alternative treatment plans, and other information in a timely manner and obtain his express consent.*

*Where it is impossible or* **inappropriate** *to do so, the medical staff shall explain it to the patient’s close relatives and get their express consent.*’

The determination of ‘inappropriateness’ in such cases is subjective and value laden. Generally, it encompasses situations in which disclosure to the patient might lead to negative outcomes. On the one hand, patients in palliative care are often physically and mentally fragile, and the burden of processing complex medical information may cause needless exhaustion. Such exhaustion is considered an ‘*unnecessary burden*’ that can be avoided by not initiating the discussion ([28], p.74). On the other hand, the content of the information in palliative care - malignant diagnoses, limited life expectancy, and deteriorating trajectories - is deemed potentially harmful, as it may induce suffering or distress in the patient. This emotional toll might, in turn, compromise the effectiveness of treatment, resulting in further harm [28]. Consequently, it is common practice in palliative care wards to redirect most, if not all, medical information to the family, and to seek informed consent from them instead of the patient. In this way, Chinese families are legally bound to participate in decision-making and to provide informed consent for patients receiving palliative care.

However, this legal necessity conflicts with the epistemological foundations of the four-principles approach. In Beauchamp and Childress’s theory of autonomy, one of the three prerequisites for an autonomous choice is the absence of ‘*controlling influences that determine their [the patient’s] action*’ ([2], p.104). Within a patient-centred care model, it is considered an ethical obligation for HCPs to ensure that patients can make decisions without undue interference.

In their further discussion, Beauchamp and Childress [2] identify coercion and manipulation as key forms of controlling influence. In the Chinese context, family-led decision-making is driven by the moral duty of filial piety and thus does not generally constitute coercion at the motivational level. Yet, having the family make decisions for the patient could be classified as manipulation, defined as ‘*sway*[ing]* people to do what the manipulator wants by means other than coercion or persuasion*’ ([2], p.139). While some degree of persuasion may occur in family discussions, withholding ‘inappropriate’ information and overturning patient decisions align closely with the principlist notion of manipulation. From the standpoint of the four-principles approach, this suggests that family-led decision-making in China should be excluded from caregiving to safeguard the patient’s autonomy.

This exclusion, however, is ill-suited to the Chinese legal framework, which mandates family participation as the source of informed consent. Hypothetically, if Chinese HCPs were to apply the four-principles approach strictly and exclude the family from decision-making, three possible scenarios might arise:

1. **Disclosure to the Patient Alone**: HCPs would have to inform the patient of ‘inappropriate’ information, causing avoidable harm and directly contradicting both non-maleficence and Chinese law [the Civil Code, Article 1219].
2. **Nondisclosure to Both Patient and Family**: To avoid potential harm and uphold principlist duties, HCPs might refrain from informing either party. However, this would prevent them from obtaining any valid informed consent, forcing the cessation of care.
3. **Continued Care Without Valid Informed Consent**: If HCPs proceed without informed consent, they risk breaching the law and facing legal repercussions.

Each of these outcomes is ethically problematic or even unlawful, thus undermining the objective of using the four-principles approach to resolve ethical dilemmas. In this sense, applying the four-principles framework in the Chinese palliative care context not only fails to offer practical utility but may also obstruct clinical practice and lead to undesirable results.

## Conclusions

In sum, the direct application of the four-principles approach within the Chinese palliative care context reveals fundamental tensions between Western bioethical assumptions and the legal, cultural, and moral landscape shaped by Confucian traditions and Chinese law. For Chinese HCPs, the simultaneous accommodation of both patient autonomy and the family’s moral significance in palliative care practice remains an unattainable goal.

In its current translation, the four-principles approach does not sufficiently address the moral significance of the family and Familism in Confucian ethics within a *Confucian* cultural context. This approach retains its individual-centred core, which primarily excludes the family from formal caregiving and related ethical considerations. This exclusion undermines the duty of familial involvement in caregiving within the Chinese mainland. By neglecting the family’s crucial role, the four-principles approach reaches an impasse. Rather than facilitating ethical decision-making, the framework instead complicates the practice of palliative care in China by generating competing ethical obligations and potentially unlawful consequences, thereby contradicting the fundamental purpose of the four-principles approach. Furthermore, the current coping mechanism proposed by Chinese HCPs remains on a shaky ethical foundation, whose validity cannot be soundly justified.

To progress, future efforts to apply the four-principles approach must incorporate the local social, moral, and legal foundations of Chinese mainland. Empirical evidence demonstrates that the family in China is so far treated as a formal stakeholder at both practical and ethical level. If principlism is to remain integral to the education - and ultimately the practice - of palliative care professionals in China, it must develop a comprehensive and well-founded response to the role of the family, and the Confucian prima facie principle of filial piety, both of which are deeply embedded in and underpin the Chinese context.

## Supplementary Information

Supplementary Material 1.

Supplementary Material 2.

## Data Availability

The dataset generated and analysed during the current study are not publicly available since the study is still ongoing. After the project is finalised, the data will be made available from the corresponding author on reasonable request.

## Footnotes

1 At the suggestion of one of the peer reviewers we would like to clarify that the four principles were listed in this order by the participants. While the participant presented the four principles in a slightly different order from the traditional principlist framework, the content remained complete. This articulation of the four principles reflects the Chinese HCPs’ understanding of Principlism.

2 The laws and regulation which mandates the legal responsibilities of the family in medical practice include

- • Law of the People’s Republic of China on Basic Medical and Health Care and the Promotion of Health
- • Law on Doctors of the People’s Republic of China
- • Regulation on the Administration of Medical Institutions
- • Regulation on the Prevention and Handling of Medical Disputes.
