# Supplementary material for: Translation of bioethics across cultural borders: exploring the adoption of the four-principles approach in palliative care provision on the Chinese mainland
Source: BMC Palliat Care. 2025 Apr 10;24:100. doi: 10.1186/s12904-025-01733-2 (PMC11984173; doi:10.1186/s12904-025-01733-2)
Supplement: Supplementary file 1 — Supplementary Material 1. [file 12904_2025_1733_MOESM1_ESM.pdf]

Participant Information Sheet

Project title: *Exploring Everyday Ethics in Palliative Care in China: A Qualitative Empirical Bioethics Study*

Dear participant,

This is Shengyu Zhao, a second-year PhD student at the Centre for Ethics in Medicine, University of Bristol in the United Kingdom. I would like to invite you to take part in my research project – *Exploring Everyday Ethics in Palliative Care in China*. The project is part of the Population Health Sciences PhD programme hosted by the Bristol Medical School, University of Bristol, and funded by the Wellcome Trust. It aims to explore how Chinese healthcare professionals understand and address ethical challenges in their practice of palliative care. The project has been reviewed and approved by the Faculty Research Ethics Committee of Faculty of Health Sciences, University of Bristol. The approval reference number is 12319.

Before you decide whether to participate, I would like you to understand why the research is being conducted and what it would involve for you. Talk to others about the study if you wish. Please ask me questions if anything is unclear.

What is this project about?

This project plans to investigate Chinese palliative care professionals' experiences of ethical issues and challenges arising in their work.

'Ethical issue' is a rather broad term. You may, for example, understand it as an incident, situation or experience that

- You find difficult to resolve in a satisfactory way;
- makes you feel uncomfortable or that something is wrong;
- makes you feel things should not be this way.

You may have such feelings while interacting with a patient, a family, a caregiver or a colleague.

We would like you to talk about such experiences, including what it was, how you felt at that time, how you navigated through it and if you have any reflections on it.

What does this project aim to do?

The project aims to explore what ethical issues Chinese healthcare professionals have encountered and how these issues are responded to. This project has three fundamental goals:

- To investigate and collect empirical evidence about the ethical issues – including ethical challenges – that have been encountered by Chinese healthcare professionals;

- To find out how existing education and training schemes address the ethical challenges identified;
- To propose suggestions and recommendations based on my analysis that can help to improve the effectiveness and specificity of training and education.

Why am I invited?

Up to 30 professionals who have experiences related to palliative care provision will be invited to take part into this research.

You are invited to take part if you are:

- a medical professional (e.g., doctor, therapist, nurse, etc.) who works in any publicly or privately funded hospital, and from the department of geriatrics, oncology, or palliative care; or
- a public health professional (e.g., general physician) who is not hospital-based, but works in a community clinic, hospice or care home; or
- other supportive roles in a palliative care team, e.g., medical social worker, volunteer, psychological counsellor, etc.

And you are working in

- a geriatric palliative care ward,
- a palliative care department,
- a palliative care team in a hospital,
- a hospice,
- a care home.

And you can

- speak Mandarin or English fluently;
- provide consent to participation.

What will happen if I take part?

If you agree to participate you will be invited to a one-to-one interview with a researcher. The interview will be held in a hybrid form. It can be held in person if you prefer and the circumstance allows. However, if it is due to the COVID-19 restriction policies or your preference, the interview can also be arranged online using the application *Tencent Meeting*.

If the interview is in person, the location of the interview will be arranged either within or nearby your workplace for the convenience of commuting. Commonly, the interview will be conducted within a closed and private room arranged by your host institution in which only you and the interviewer will be present. Otherwise, the researcher will rent a meeting room to establish a private environment. There will only be you and the interviewer present in the room.

If the interview is held online, the researcher will send you an invitation link on the platform of *TenCent Meeting* (VooV Meeting) a day before the interview. You will be asked to find a suitable place, preferably a private environment where you will not be interrupted during the interview. We will spend the first 5 minutes of interview to test the internet connection, the camera, and the microphone from both sides.

Please note since this platform is rarely used in the UK, *TenCent Meeting* (VooV Meeting) is not a university approved platform for now. All online interviews are therefore subject to the

terms of service and privacy policy of the platform. The University is not responsible for the operation of the platform and cannot guarantee its security.

Why TenCent Meeting (VooV Meeting)?

TenCent Meeting (VooV Meeting) is one of the most widely used online video conferencing platforms in China. Most participants are expected to understand how to access and operate this platform. Therefore, choosing *TenCent Meeting* (VooV Meeting) is convenient for both the researcher and the participant.

Other university approved platforms include Teams and Zoom. Teams is rarely reported to be used in the hospital and other medical institutions. Therefore, choosing Teams means certain amount of participants are not familiar with the settings and operation of this platform, which may significantly impact the quality of the interviews.

Zoom is not selected due to technical reason. Since May 2021, Zoom shut down most of the functions for private accounts in the area of Mainland China. In addition, it has been frequently reported that users in Mainland China with a private account often have access issues and broken connections. These problems would cause severe damage to the quality interview, hence Zoom is not considered for this project.

What does an interview involve?

The interview will last for approximately an hour. However, the length of interview is flexible and dependent on how the conversation goes.

During the interview, we will talk about your personal experiences of any ethical issues or challenges you have encountered in your work, including but not limited to

- what are your experiences?
- how do you feel about such experiences?
- do you have any reflections on such experiences?
- have you received any training which aims to assist you to manage such scenarios?
- how do you feel about the training?
- do you think anything can be changed/improved to make the training more effective?

Whether in-person or online, the interview will be recorded using an encrypted device to preserve your privacy.

What happens next?

The interview recording will be transcribed by a University approved transcriber. The transcript will be checked and anonymised. This means all your personal information will be removed and you will not be identifiable. The original audio recording will also be deleted after transcription.

Are there benefits in taking part?

There will be no direct benefits to taking part, although you may find it beneficial to share your views and experiences.

As a form of appreciation, there will be a small gift of University of Bristol branded souvenirs and a thank-you letter provided if you take part in an interview.

Will my expenses be reimbursed?

As interviews are planned to be held in or nearby your workplace or online, we do not think you will incur any extra expense taking part, so there will not be any travel reimbursement. However, if an interview in your workplace is not possible and another location is chosen, your travel costs will be reimbursed. If circumstances apply, please provide the proof (e.g., receipts) of relevant costs to the researcher on the day of interviews.

Are there risks in taking part?

This research is considered minimal risk. Nevertheless, there are potential minor negative impacts dependent on your experiences.

You may feel upset or distressed while talking about challenging experiences. If you express or exhibit any distress or discomfort, the interview will be paused. You will be asked if you wish to resume and given the opportunity to terminate the interview if you wish. You will be free to pause or stop the interview at any time.

As the interview is designed to discuss your previous experiences, you might disclose confidential, identifiable patient information. Any details of this sort will be removed from the interview transcript or changed so that you and the patient remain anonymous. No identifiable information about you or your patients will be published in the subsequent outputs (primarily, a PhD dissertation, an outcome report for participants, written publications and academic presentations).

What will happen if I don't want to carry on with the research?

It is up to you to decide whether you wish to participate in the project.

The researcher will describe the study and go through this information sheet with you before you participate and answer any questions you might have. If you agree to take part, the researcher will then ask you to sign a consent form.

You are free to withdraw at any time from the interview, without giving a reason. Recording will cease the moment you decide to stop or withdraw your participation.

You are entitled to withdraw from the study at any point before the anonymisation of your data, which will be 7 days after data collection, without giving a reason. Once you decide to quit, your data will be removed from researcher's database. There will not be any impact on your future work or life.

Will my personal information be kept private?

Personal information is anything that might be recognised about you, like your name and contact details. This information will be kept private. No private matters that can be linked to you personally will be shared with anyone outside the researcher and her academic supervisors.

The reasons any private matters could be shared are

1) If a court orders this information to be released because it relates to a possible crime: this is very unlikely to happen.

2) If the researcher has concerns that the information disclosed during interviews may negatively impact your or your patient(s)' safety and wellbeing.

If the researcher identifies any concerning information in your interview, she will firstly report to her academic supervisors to confirm and assess the level of harm. If potential harmful consequences are evaluated and confirmed, the researcher will contact your department head and report the incident. Your supervisor/manager will notify you what will happen next.

In any other situation, we will not mention your name, or information which will be recognised by other people as being about you, in any results from the research.

How will my information be stored?

Private information about you (like your name and contact details) will be stored securely, in either locked cabinets or on password-protected areas of computers and cloud drives. We will follow the University of Bristol's 'research governance policy'.

Will my research information be shared?

Your 'research information' is the things you tell the interviewer about your experiences. These things will be 'anonymised', which means they can't be recognised as being about you personally.

Written reports from the research may include some examples of what you said in interview. Nothing that could be recognised about you personally will be quoted.

Anonymised data (data where private information has been taken out) may be shared with other researchers doing research in this area. Any requests from other researchers to look at the data gathered will be carefully checked and will need approval by the University of Bristol.

At the end of the study, the anonymised data will be made "Open Data". This means that it will be stored in an online database so that it is publicly available.

What is open data?

Open data means that data are made available, free of charge, to anyone interested in the research, or who wishes to conduct their own analysis of the data. We will therefore have no control over how these data are used. However, all data will be anonymised before it is made available and therefore there will be no way to identify you from the research data.

Why open data?

Open access to research findings and access to data is considered best research practice and is a requirement of many funding bodies and journals, including the funder of this project – the Wellcome Trust. Sharing data helps to maximise the impact of investment through wider use, and encourages new avenues of research.

What will happen to the results of the research?

The outcomes of this research will be primarily used to produce the researcher's PhD degree thesis. They may also be used in subsequent publications in medical and scientific journals.

The research results may also be presented at scientific conferences in order to circulate them as widely as possible.

Please be assured that any of your personal information, including but not limited to your name, age, position, institution, etc. will be removed or anonymised within any thesis, publications, and/or presentations.

How can I hear about the research outcomes?

After the interview concludes, you will be asked if you would like to be informed of the results of the study, and your response will be recorded.

If you respond positively, you will be sent either a copy of my thesis or a summary of results or both (based on your preference) after the research is completed.

Who can I get in touch with if I have any questions?

If you would like to know further information in relation to the study and have any questions regarding the project, please do not hesitate to get in touch with the researcher via sz15505@bristol.ac.uk or +44 (0)117 455 7689. Please note this is a UK number, if you call or text from Mainland China, additional costs for international roaming may be incurred. For further details please consult your mobile operator.

If you have any concerns related to your participation in this study or you would like to make a complaint, you can contact the Faculty of Health Science Research Ethics Committee, via the Research Governance Team, research-governance@bristol.ac.uk.
