# Supplementary material for: Translation of bioethics across cultural borders: exploring the adoption of the four-principles approach in palliative care provision on the Chinese mainland
Source: BMC Palliat Care. 2025 Apr 10;24:100. doi: 10.1186/s12904-025-01733-2 (PMC11984173; doi:10.1186/s12904-025-01733-2)
Supplement: Supplementary file 2 — Supplementary Material 2. [file 12904_2025_1733_MOESM2_ESM.pdf]

Exploring Everyday Ethics in Palliative Care in China: A Qualitative Empirical Bioethics Study

Interview Topic Guide

Prior to the dialogue described here, the information sheet will be given to the participant and the contents discussed. Participants will be given the opportunity to ask any further questions before they are asked if they consent to the interview taking place. Participants will sign two consent forms, retain one and send a copy to the research team.

This is not intended to be a precise script. The following questions and dialogue are illustrative of the kind of questions which will be asked at interview.

First of all I'd like to thank you for taking the time to participate in this project.

I am interested in finding out what different people think about ethical issues and challenges arising in their practice of palliative care, and how they deal with these. Your views are valuable to me, and there are no right or wrong answers. To explore this, I would like to discuss your experiences, feelings and your opinions about dealing with ethical issues.

You don't have to answer any question that you would rather not answer, so if I ask questions that make you feel uncomfortable at any time, please let me know, and we'll move on. Please also bear in mind that we can pause or stop the interview at any stage if you need a break, get fed up or don't want to carry on. I would expect the interview to last an hour, but we can go at whatever pace feels right for you.

Section I Warm up

- Have you ever taken part in a research interview before? How did you find it?
- How did you hear about this study?

Section II Experience in Palliative Care

I'd like to start by talking about your experience in palliative care.

- *Can you tell me a bit about yourself?*
  - *Age, gender*
  - *Education level*
  - *Institution*
  - *Specialty*
  - *Years of experience*
  - *Current position/occupation*
- *What brought you to palliative care?*
- *What are the major services provided in your institution?*
- *What's your role in palliative care? what do you usually do on daily basis?*
  - *What sorts of patient do you encounter?*

Section III Experiences of Ethical Issues

I would like to talk about any ethical issues, challenges or problems that you have encountered when providing palliative care.

- *What sorts of issues (challenges, problems, difficulties, etc.) do you usually encounter?*
- *What is the issue (challenge, problem)?*
  - *Who finds it challenging? You as a professional? Your colleagues? The patient's family? The patient? The community? The hospital/institution?*
- *Which part do* ***<u>you</u>*** *think is the most challenging to deal with? And why?*
  - *Which aspects/details of this problem make you feel uncomfortable? In what way(s)?*
- *Are there any other ethical issues commonly arising in your everyday practice?*

Section IV The Solutions and Responses

Regarding the case(s) you just mentioned, I would like to know how you managed it.

- *Who was involved in the problem-solving process? Who contributed?*

    - *Do you think anyone should or shouldn't be involved in this process? Why?*
- *What kinds of procedures or processes do you normally go through?*
    - *Is this what happened in the case you mentioned? If not, what happened there? And why was it different?*
    - *Did you encounter any problems when you tried to sort this case out? Anything went wrong? Why do you think it's a problem/it went wrong?*

While you were thinking of a solution,

- *Regarding the case, did you come up with a solution?*
- *Do you think this was an appropriate solution? Why/not?*
    - *If it was not appropriate, what do you think would have been an appropriate solution? Why? And why wasn't the more appropriate solution adopted at the time?*
    - *Do you feel wrong whatever you do? What made you feel so?*
- *What did the patient and the family think of the solution(s)?*

Whenever you attempt to solve these sorts of issues,

- *What sorts of support are you able to draw upon? Education? Training? Guidance? Ethics support (committee)? Other?*
    - *Do you find it/them helpful? Why? Why not?*
- *What sorts of support do you think should be available in the future? More training opportunities? Guidance e.g. on a national level?*
    - *Why do you think they can be helpful?*

Section V Conceptualisation

We've been talking about *ethical* issues or challenges, and I'm interested in the meaning of terms like 'ethics' or 'ethical issues'. Therefore, I would like to know …

- *How do you understand these phrases?*
- *What makes something an ethical issue compared with other issues (financial, artistic)? What do you consider to be the key features?*

Section VI Closing

Thank you for all your answers. I have no further questions to ask, but is there something you would like to add?

- *How did you feel about the interview?*
- *Would you like to know what happens next with the information you've given?*

Interview End

Obviously we are in a pandemic at present, which is limiting public events. However, when I am able to do so, I hope to give talks at the University of Bristol and/or online where members of the public, academics, and clinicians can hear more about how this research is progressing, and any emerging results. Would you like me to contact you with the times of these talks when they are coming up?

(Interviewer to complete) ☐

Once the study is completed, I'll be preparing a report with the study results for the people who've given their help. Would you like me send you a copy of this report?

(Interviewer to complete) ☐

Thank you for taking the time to give this interview.
